# Dress syndrome, gabapentin (GB) and autografts

**DOI:** 10.1186/1939-4551-8-S1-A166

**Published:** 2015-04-08

**Authors:** Carolina Aranda, Marcia Costa, Paula Meireles, Eduardo Natel, Aline Simplicio, Andrea Ostaszewski, Diego Varella, Gustavo Falbo Wandalsen, Priscila Feliciano

**Affiliations:** 1 Hospital Do Servidor Publico Municipal De São Paulo, Brazil; 2 Universidade De São Paulo, Brazil; 3 Federal University of Sao Paulo, Brazil; 4 Brazilian Society, Brazil

## Background

The DRESS syndrome (drug rash with eosinophilia and systemic symptoms) is an adverse drug reaction with systemic features, which mainly includes a severe rash, fever, lymphadenopathy, hepatitis and hematological abnormalities. The mortality rate is approximately 10%. The aim of this study is to relate DRESS, GB and autografts.

## Methods

Female, 64 years old, diabetic, hypertension and chronic obstructive arterial disease (COAD) for over 5 years. The illness had worsened, with obstruction of the left anterior femoral tibial artery. First, we tried conservative treatment with angioplasty and GB for pain control. Contralateral saphenous vein graft had been done because the first procedure was not effective.

## Results

After 28 days the use of GB and 20 days of grafting, she had morbilliform rash, fever (39C), cervical and inguinal adenopathy. CBC was performed: hemoglobin 10,3g/dl, leukocyte 14,900/mm³, neutrophils 58.9%, eosinophils 16.4% (2,443), lymphocytes 10% and monocytes 3.1%. Platelets were normal. ALT 200 U/L (up to 55), AST 180U/L (up to 34), creatinine 1.8 mg/dl (up to 1). Previous examinations showed no changes except for leukocytosis with a left shift. As European Registry of Severe Cutaneous Adverse Reactions (SCAR) to Drugs and Collection of Biological Samples, the patient had score 6 (definite case). GB was removed and the patient was treated with methylprednisolone 120 mg / day IV for 3 days. Thereafter, the medication was continued at a dose of 60mg/day for 5 days. After 72 hours, the patient was afebrile with improved lymph node, the rash, renal and hepatic parameters. Gradual withdrawal of corticosteroids had done because the patient had significant eosinophilia (≥ 1500/mm³), prednisone 30mg for 7 days, then 20 mg for 7 days, 10mg for 7 days and maintained 5mg for 20 days. After three months, the patient showed eosinophilia but no acute relapse of the condition.

## Conclusions

One case of DRESS related to GB after autologous graft is reported in the literature. No registration GB related DRESS is in SCAR. The pathophysiology of this disease is not fully understood, but studies on the immune response after surgery, anesthesia and grafts are also needed, especially their relationship with inflammation and eosinophils and lymphocytes activation that may contribute to adverse drug reactions.

## Consent

Written informed consent was obtained from the patient for publication of this abstract and any accompanying images. A copy of the written consent is available for review by the Editor of this journal.

